# Screening and evaluation of key technologies for non-bioartificial liver care: an empirical study

**DOI:** 10.3389/fmed.2024.1459428

**Published:** 2025-01-07

**Authors:** Yunzhi Zhang, Han Zhang, Ling Luo, Zhen Liu, Anlin Liu, Lin Shi, Luwen Liang, Jing Zhao, Pu Chen, Yanli Yang

**Affiliations:** ^1^Department of Infectious Diseases, The Second Affiliated Hospital of Chongqing Medical University, Chongqing, China; ^2^Department of Nephrology, Children’s Hospital of Chongqing Medical University, Chongqing, China; ^3^National Clinical Research Center for Child Health and Disorders, Ministry of Education Key Laboratory of Child Development and Disorders, Chongqing Key Laboratory of Pediatrics, Chongqing, China

**Keywords:** non-bioartificial liver, nursing technology, key technology, empirical research, Delphi method, hierarchical analysis method, TOPSIS method

## Abstract

**Introduction:**

To identify key technologies within non-bioartificial liver (NBAL, an extracorporeal support system that temporarily replaces some of the liver’s functions) nursing to offer guidance for clinical practice. In the context of NBAL nursing, key technologies are crucial for successful implementation of artificial liver treatment, ensuring patient safety, and enhancing nursing quality. A review of both domestic and foreign literature revealed that studies on NBAL nursing technology are lacking and that the key technologies for NBAL nursing have not been clearly identified.

**Methods:**

Using empirical research methods to collect and analyze data. First, the on-site survey method and literature research method were used to create a preliminary screening list of key technologies for NBAL care. Next, the focus group discussion method was used to establish the screening principles and evaluation indicators for these key technologies. Then, a two-round Delphi study via e-mail correspondence was used to screen and determine the key technologies for NBAL care. Finally, the analytic hierarchy process (AHP) and the technique for order preference by similarity to ideal solution (TOPSIS) comprehensive evaluation method were applied to evaluate these key technologies for NBAL care.

**Results:**

Seventeen key technologies for NBAL care were identified. These include three basic nursing technologies, seven operating techniques, three items for treatment process monitoring technology, two items for health education, and two items for complication prevention and treatment technology.

**Conclusion:**

This study identified key NBAL nursing technologies, offering a systematic guide to enhance clinical practice. These technologies improve treatment safety, efficacy, and nursing standards, laying a foundation for NBAL care advancement.

## 1 Introduction

Liver failure (LF) is severe liver damage caused by various factors, impairing essential functions, such as synthesis, detoxification, metabolism, and biotransformation, and manifesting as jaundice, coagulation dysfunction, hepatorenal syndrome, encephalopathy, and ascites (1), with a high mortality rate of up to 70% (2). Non-bioartificial liver (NBAL) is a critical adjunct therapy for acute or chronic liver failure (3). It includes various treatment modalities, such as plasma exchange (PE), hemodialysis filtration, hemoperfusion (HP), plasma bilirubin adsorption (PBA), and the double plasma molecular adsorption system (DPMAS), which are categorized based on therapeutic principles and indications. Despite variations in equipment configuration and operational details, these modalities share the common goal of detoxifying the blood and compensating for compromised liver functions through adsorption, dialysis, and filtration of harmful substances and toxins (4, 5). NBAL not only facilitates liver function recovery but also improves patient outcomes, reduces complication risks, and serves as a bridge to liver transplantation or other alternative therapies (6, 7).

NBAL nursing work is an important component of artificial liver treatment. The “Expert Consensus on the Clinical Application of Artificial Liver Blood Purification Technology (2022 Edition)” (8) and “Guidelines for the Treatment of Liver Failure with Non-Biological Artificial Livers (2016 Edition)” (9) both emphasize the significance of safe and effective professional nursing technology throughout the treatment process. With the evolution of modern medical concepts and nursing models, nursing technology has transformed beyond just motor skills to include experiential, substantive, and knowledge-based skills, thus playing an indispensable role in clinical practice (10). Empirical technology involves nursing experience and skills; substantive (physical) technology involves medical equipment and medications; and intellectual technology involves technical knowledge, nursing processes, standards, and health education.

The NBAL process is evidently a systematic procedure that encompasses various aspects of nursing technology. The following exemplify essential nursing technologies: environmental and instrument disinfection before the NBAL treatment, patient assessment and evaluation, heparinization of extracorporeal circulation pipeline during treatment, instrument and equipment parameter setting and alarm processing, blood product infusion, medical waste disposal after treatment, maintenance of vascular access, post-extubation care, basic peri-treatment patient care, vital sign monitoring, early warning of nursing risks, observation and treatment of complications, diet and activity guidance, psychological care, and nursing record documentation.

Among the various nursing technologies, those with characteristics like importance, leadership, applicability, transformation, and safety are considered key technologies (11). Key technologies are the “bottleneck” technologies that limit the development of a specific industry or field and hold significant economic and social value. For instance, key medical and health technologies are essential for enhancing the quality of people’s health (12). Notably, new technologies may not always be key technologies, and significant technologies may not necessarily be classified as key technologies. The determination of a technology as a key technology is influenced by the goals of application, the stage of application, and the overall environment (13). Despite the diversity in NBAL models, the pivotal role of core nursing techniques in safeguarding treatment efficacy and patient prognosis remains uniform. In summary, within the realm of NBAL nursing, the mastery of key technologies is indispensable for the seamless execution of artificial liver therapies, assurance of patient safety, and advancement of nursing standards. Consequently, this subsequent discourse will concentrate on the essential nursing methodologies that are applicable to all NBAL configurations.

Current research lacks a comprehensive, standardized scientific framework for NBAL nursing techniques, leading to the absence of clear identification and emphasis on key nursing technologies. This deficiency results in inadequate clinical application and promotion of NBAL, with the complexity of high-skill, risk-associated technologies being further overlooked. A review of the existing literature reveals a significant gap in NBAL nursing technology studies, with key methodologies remaining ill-defined. The present study aims to delineate essential nursing technologies in NBAL care, focusing on the staff’s use of instruments, knowledge, skills, and attitudes. These technologies are pivotal for mitigating complications, optimizing treatment outcomes, enhancing quality of life, and reducing mortality. By identifying these technologies, we aspire to bridge the gap between clinical practice and theory, thus providing clinical guidance and contributing to the establishment of a systematic, standardized, and scientifically robust framework for NBAL nursing.

## 2 Materials and methods

This study primarily used empirical research methods to gather data.

### 2.1 Developing a preliminary list of key NBAL nursing technologies for screening

#### 2.1.1 On-site survey method

This study used a qualitative research approach through an extensive on-site investigation at the Artificial Liver Treatment Center, Department of Infectious Diseases, Second Affiliated Hospital of Chongqing Medical University. We carefully observed the NBAL treatment procedures executed by nursing staff, meticulously documented each step, and compiled a preliminary list of key NBAL nursing technologies for subsequent screening.

#### 2.1.2 Literature research method

We conducted a systematic literature search across Chinese databases (China National Knowledge Infrastructure, VIP, and Wanfang database) and international databases (PubMed, Medline, Elsevier ScienceDirect, and Web of Science). Our search used keywords relevant to the NBAL nursing technology, including “artificial liver,” “NBAL,” “plasma exchange,” and related terms. The objective of this comprehensive search was to compile a preliminary list of key NBAL nursing technologies for further analysis and evaluation.

### 2.2 Determine the principles and evaluation indicators for NBAL nursing key technology screening

Focus group discussion methodology was used to establish the principles and evaluation indicators for screening of NBAL nursing key technologies. The group comprised nine members, each with over 10 years of experience in the medical or nursing field at the artificial liver treatment center. This group included one chief physician, two chief nurses, four supervisory nurses, and two nurse practitioners, with a composition of one doctor, five masters graduates, and three undergraduates.

### 2.3 Screen and evaluate key technologies in NBAL nursing

#### 2.3.1 Delphi expert consensus method

##### 2.3.1.1 Expert selection criteria

1. Minimum of 10 years of clinical experience in diagnosing and treating liver disease or working in NBAL nursing
2. Bachelor’s degree or higher
3. Intermediate professional title (supervisor nurse) or higher
4. Experience in scientific research
5. Demonstrated interest and active participation in this study

##### 2.3.1.2 Contents of the expert letter questionnaire

1. The questionnaire provided a description of the research background on this topic to the experts as well as an explanation of the research purpose and tasks.
2. Questions in the main body of the questionnaire pertained to the importance and complexity of technology recognized by experts and were evaluated by the experts on a 5-point Likert scale. The primary indicators were rated based on importance levels: very important (5 points), important (4 points), generally important (3 points), not important (2 points), and very unimportant (1 point). Secondary indicators were rated on a 5-point Likert scale considering both their importance and complexity. Higher scores indicated higher importance and complexity. Each item had a column for modification comments where experts could provide any suggestions for modifications or make any additions.
3. The experts’ basic information table included responders’ sex, age, and education, among other parameters. In addition, the experts’ authority level was assessed based on their self-evaluation of familiarity with this field. Familiarity was rated as very unfamiliar, unfamiliar, general, familiar, and very familiar, with assigned values of 0.2, 0.4, 0.6, 0.8, and 1.0, respectively. The basis for judgment included practical experience, theoretical analysis, reference to domestic and foreign literature, and intuitive judgments, with the influence level categorized as large, medium, and small, each assigned different quantitative values.

##### 2.3.1.3 Questionnaire distribution

The researchers administered the questionnaire via email in March 2022, and responses to each round of questionnaires were collected within 2 weeks.

##### 2.3.1.4 Indicator screening criteria

In the first round of expert consensus feedback data, technical indicators with an average value of <3.5 points and a coefficient of variation of >0.25 at any evaluation level of importance and complexity were included in the sequence to be deleted. In the second round of feedback results, if the technical indicators in the sequence to be deleted had a mean value of <3.5 points and a coefficient of variation of >0.25 at any evaluation level of importance and complexity, they were deleted. Controversial indicators were discussed within the focus group, and expert opinions were combined. After the first round of questionnaire responses was collected, the results of the first round of the expert consensus were summarized, sorted, and analyzed according to the indicator screening standards. Expert opinions and suggestions for modification were discussed, and the corresponding indicators were modified to form the second round of expert consultation inquiry form, along with a summary of the first round of experts’ revision opinions and scores for each indicator. The expert consensus inquiry was terminated when the opinions of experts tended to converge.

##### 2.3.1.5 Statistical analysis

We used Microsoft Excel 2019 (Redmond, WA, USA) to create a database and organize and summarize the data. Then, we used SPSS 26.0 software (SPSS Inc., Chicago, IL, USA) to perform statistical analysis on the experts’ basic situation, enthusiasm, authority, and coordination.

#### 2.3.2 Analytical hierarchy process and TOPSIS comprehensive evaluation method

The analytic hierarchy process is a multi-objective decision-making analysis method that combines quantitative and qualitative analysis. It was proposed by an American scientist, T.L. Saaty, in the 1970s (14). In this study, the first-level indicator used the eigenvector method for calculation. Its basic principle is to decompose complex decision-making problems and quantitatively analyze the importance of each level. These levels can be divided into the highest level (purpose layer), the middle level (criteria layer), and the lowest level (plan layer). In this study, the technique for order preference by similarity to ideal solution (TOPSIS) comprehensive evaluation method was used to screen and evaluate the secondary indicators of NBAL key nursing technologies. TOPSIS is a sequential optimization technology based on similarity with the ideal solution, and it is a common method for multi-objective decision-making analysis in systems engineering. The basic idea of this method is to find the optimal solution and the worst solution among the limited solutions, which are represented by the optimal vector and the worst vector, respectively. Then, the relationship between the evaluation objects and the distance between the optimal solution and the worst solution was calculated to determine the relative closeness of each evaluation object to the optimal solution, which serves as the basis for evaluating their merits (15).

The basic steps of TOPSIS were as follows. The first step was to align the indicators with the trend. As NBAL nursing technical indicators were all high-quality indicators, there was no need to perform same-trend processing.

The second step involved normalization processing. After experts scored the indicators based on the two evaluation dimensions of importance and complexity, they could calculate the mean importance and mean complexity. Then, they performed normalization processing according to the normalization Equation 1 below.

The third step was to determine the optimal solution Z+ and the worst solution Z−. In the normalized vector, the maximum of each screening index represented the optimal vector, whereas the minimum of each screening index represented the worst vector.

The fourth step involved calculating the distance D+ between each technical indicator and the optimal solution and the distance D− between each technical indicator and the worst solution according to Equations 2, 3. The acceptance degree Ci between the evaluation object and the optimal solution was then calculated according to Equation 4 (The value of Ci ranges between 0 and 1. The closer its value is to 1, the closer the evaluation object is to the optimal solution, and vice versa for the worst solution.).

In this study, NBAL nursing technical indicators were sorted according to their Ci values. Higher Ci values indicated greater importance and complexity of the technical indicator, resulting in a higher ranking.


(1) $$Z_{ij}=\frac{a_{ij}}{\sqrt{\sum_{i=1}^{n}a_{ij}^{2}}}$$

(2) $$D_{i}^{+}=\sqrt{\sum_{j=1}^{m}{\left(a_{ij}^{+}-a_{ij}\right)}^{2}}$$

(3) $$D_{i}^{-}=\sqrt{\sum_{j=1}^{m}{\left(a_{ij}^{-}-a_{ij}\right)}^{2}}$$

(4) $$C_{i}=\frac{D_{i}^{-}}{D_{i}^{+}+D_{i}^{-}}$$

### 2.4 Ethical principles

This study was reviewed and approved by the Ethics Committee of the Second Affiliated Hospital of Chongqing Medical University: 2020 Kelun Review No. (184), approved date Dec. 30, 2020. The research process was conducted in strict accordance with the principles of medical ethics and the requirements of the Declaration of Helsinki.

## 3 Results

### 3.1 Preliminary screening technology list of key technologies for NBAL nursing

#### 3.1.1 On-site survey method

**Figure 1:**
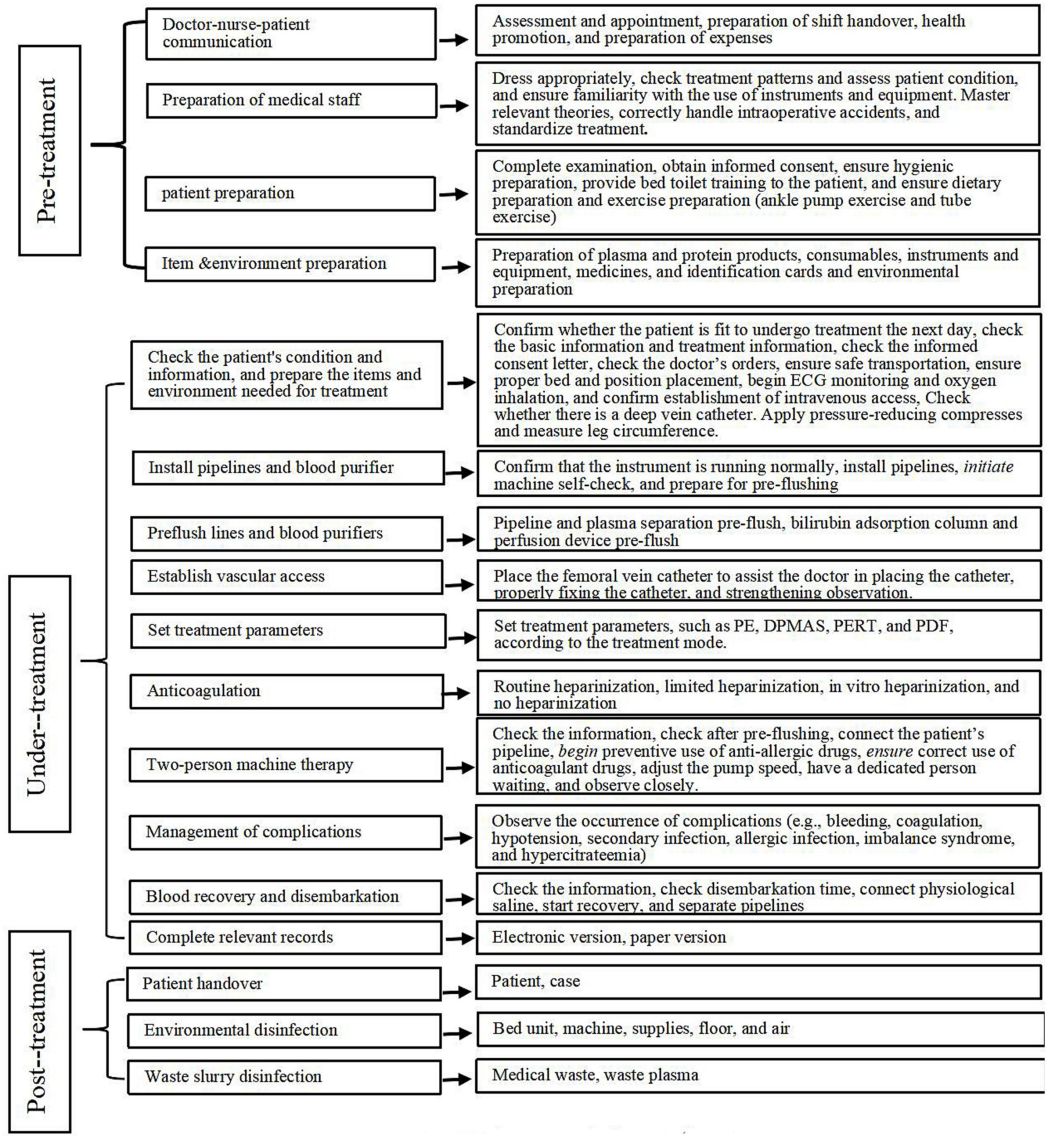
NBAL nursing workflow chart.

Following a month-long on-site investigation and subsequent data analysis, a comprehensive NBAL nursing workflow chart was meticulously documented and organized. This chart delineates the standardized protocols and identifies the essential nursing technologies critical for NBAL care, as shown in Figure 1.

#### 3.1.2 Literature research method

**Figure 2:**
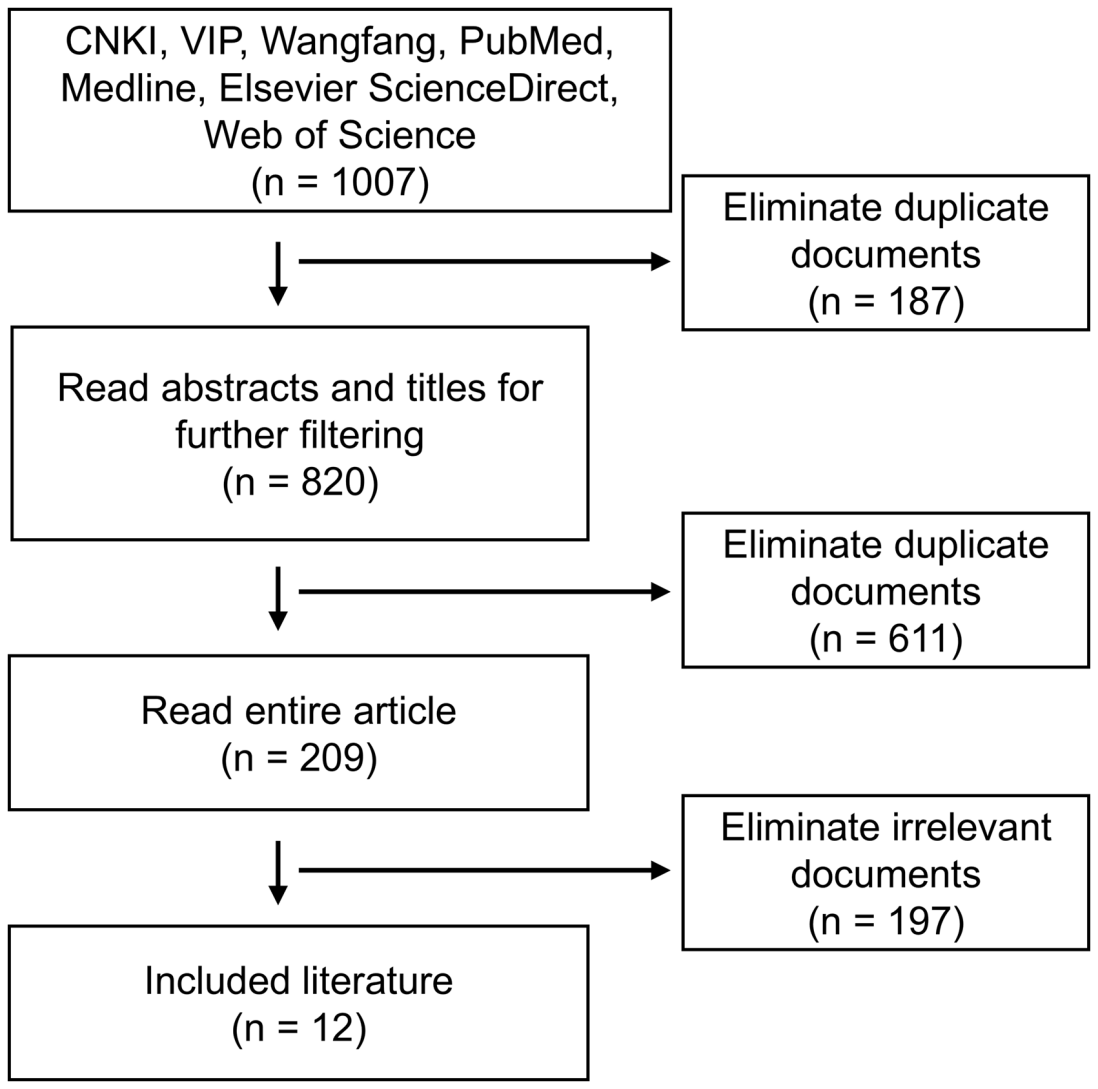
Literature screening flow chart.

The flow chart for literature screening is shown in Figure 2. Based on the 12 documents (9, 16–26) selected from the NBAL nursing workflow and literature research conducted during the on-site survey, a preliminary screening list of NBAL nursing key technologies was performed. This list includes 5 first-level indicators and 38 s-level indicators, as shown in Supplementary Table 1.

### 3.2 Determine the principles and evaluation indicators for NBAL nursing key technology screening

After discussion in the focus group, the principles for screening key NBAL nursing technologies were finally determined as follows:

1. *Scientific principle:*
  NBAL nursing key technologies play a vital role in artificial liver treatment. When formulating the initial screening list and determining evaluation indicators, we must adhere to a scientific and rigorous attitude to ensure that the selected key NBAL nursing technologies can withstand the demonstration and test of theory and practice.
2. *Demand principle:*
  NBAL nursing key technologies need to comprehensively consider subject theory and academic research needs, the urgent need for medical staff to learn skills, and the actual needs of patients and their families for treatment effects.
3. *Practicality principle:*
  NBAL nursing key technologies have strong practical application value, which can not only be applied to clinical treatment but also allow medical staff to master the application through unified training and learning.
4. *Development principle:*
  The setting of NBAL nursing technology changes with factors like disease characteristics, subject development, and technological progress, as well as with the conditions of medical units engaged in artificial livers. Therefore, the weight and ranking of NBAL key technologies will also change accordingly. This technology requires constant modification, adjustment, and improvement during clinical use.

*The evaluation indicators for key nursing technologies in NBAL are as follows:*

- *Importance:* This was determined by the nursing technology playing a crucial role perioperatively during the NBAL treatment, ensuring the safety and efficacy of the treatment.
- *Complexity:* This was determined by the difficulty of learning and mastering the nursing technology, replicating it during the NBAL treatment in clinical practice, and the level of proficiency and quality required for nursing staff to perform it safely.

### 3.3 Screening and evaluation results of NBAL nursing key technologies

#### 3.3.1 Delphi expert consensus results

This focus group used a purposive sampling method to select 20 three-level products from 17 provincial administrative regions: Hubei, Anhui, Shanxi, Guizhou, Hunan, Sichuan, Guangdong, Jiangxi, Shaanxi, Liaoning, Shandong, Chongqing, Shanghai, Xinjiang, Inner Mongolia, Heilongjiang, and Guangxi. Twenty experts from Class A hospitals participated in the consensus consultation. The basic information about the experts is shown in Supplementary Table 2.

The effective rate of questionnaire recovery in each round was 100%. The positive coefficients of experts in both rounds of consensus inquiries were 100%, indicating that experts attached great importance to and supported this research. The authoritative degree of experts in the two rounds of correspondence inquiries was 0.910 and 0.930, respectively. The degree of coordination of expert opinions was expressed by Kendall’s harmony coefficient, which ranged from 0.402 to 0.620. The values and coefficients of variation of the secondary indicator Ci in the first round are shown in Table 1.

**Table 1:** Ci values and variation coefficients of secondary indicators in the first round.

Technology	Evaluation indicator		After standardization		D+ (distance from the optimal solution)	D− (distance from the worst solution)	Ci (degree of proximity to the optimal solution)	CV	
	Importance	Complexity	Complexity	Complexity				Importance	Complexity
	Mean	Mean	Mean	Mean					
A-1 Check	4.95	2	0.179	0.094	0.124	0.055	0.307	0.01	0.5
A-2 Position management	4.3	2.45	0.155	0.115	0.106	0.042	0.284	0.165	0.305
A-3 Medical order processing	4.55	2.55	0.164	0.12	0.099	0.052	0.344	0.076	0.607
A-4 Condition Assessment	4.75	4.45	0.171	0.209	0.013	0.131	0.91	0.039	0.19
A-5 Patient handover and transfer	4	2.4	0.144	0.113	0.111	0.033	0.229	0.2	0.35
A-6 Basic daily care	3.45	2.2	0.124	0.103	0.128	0.016	0.111	0.188	0.436
A-7 Patient safety protection	4.5	3.45	0.162	0.162	0.059	0.084	0.587	0.078	0.217
A-8 Blood transfusion technology	4.8	3.6	0.173	0.169	0.049	0.095	0.66	0.054	0.206
A-9 Specimen collection	4	2.65	0.144	0.124	0.1	0.042	0.296	0.175	0.35
A-10 Prevention and control of hospital infection	4.8	3.75	0.173	0.176	0.043	0.102	0.703	0.054	0.183
A-11 Nursing document	4.55	3.35	0.164	0.157	0.063	0.081	0.563	0.076	0.277
A-12 Emotional support	4.1	3.55	0.148	0.167	0.06	0.084	0.583	0.144	0.351
B-1 Environment and equipment preparation	4.55	2.45	0.164	0.115	0.104	0.049	0.32	0.076	0.795
B-2 NBAL equipment connection technology	4.75	3.8	0.171	0.178	0.041	0.102	0.713	0.082	0.305
B-3 NBAL pipeline pre-flushing technology	4.5	3.5	0.162	0.164	0.057	0.086	0.601	0.1	0.243
B-4 Measure leg circumference	3.95	1.85	0.142	0.087	0.136	0.018	0.117	0.164	0.393
B-5 Vascular access assessment and establishment	4.85	4.65	0.175	0.218	0.005	0.141	0.966	0.026	0.07
B-6 Connection to extracorporeal circulation	4.7	3.8	0.169	0.178	0.041	0.102	0.713	0.066	0.226
B-7 Setting treatment parameters	4.7	4.15	0.169	0.195	0.025	0.117	0.824	0.066	0.223
B-8 Heparinization of extracorporeal circulation circuit	4.8	4.45	0.173	0.209	0.011	0.131	0.923	0.054	0.168
B-9 ECG monitoring and oxygen inhalation	4.4	2.85	0.159	0.134	0.087	0.059	0.404	0.077	0.29
B-10 Blood flowing back into the body from the pipeline and turn off the machine	4.45	4	0.16	0.188	0.036	0.107	0.748	0.101	0.175
B-11 Medical waste disposal	4.35	2.1	0.157	0.099	0.121	0.035	0.224	0.213	0.424
C-1 Vital signs monitoring	4.8	2.95	0.173	0.139	0.079	0.071	0.473	0.054	0.355
C-2 Puncture point status	4.65	2.9	0.168	0.136	0.083	0.066	0.443	0.092	0.272
C-3 Pipeline condition	4.65	3.9	0.168	0.183	0.037	0.106	0.741	0.113	0.228
C-4 NBAL instrument parameters	4.7	4.15	0.169	0.195	0.025	0.117	0.824	0.066	0.296
C-5 Filters and adsorbers condition monitoring	4.8	4.25	0.173	0.2	0.019	0.123	0.866	0.075	0.232
C-6 Dealing with NBAL instrument alarms	5	4.55	0.18	0.214	0.004	0.139	0.972	0	0.164
D-1 Explaining the coordination points in NBAL treatment	4.7	3.85	0.169	0.181	0.039	0.104	0.727	0.066	0.163
D-2 Inspection guidance	3.8	2.9	0.137	0.136	0.093	0.051	0.354	0.358	0.134
D-3 Safety guidance	4.55	3.35	0.164	0.157	0.063	0.081	0.563	0.076	0.187
D-4 Dietary guidance	3.75	2.9	0.135	0.136	0.094	0.05	0.347	0.183	0.238
D-5 Bed urination and defecation training	3.95	2.65	0.142	0.124	0.101	0.041	0.289	0.265	0.275
D-6 Ankle pump Exercise	4.05	3	0.146	0.141	0.084	0.058	0.408	0.16	0.167
D-7 Maintenance instructions for NBAL central venous catheters during the indwelling period	4.85	3.7	0.175	0.174	0.044	0.101	0.697	0.026	0.219
E-1 Complications of NBAL treatment	4.65	4.55	0.168	0.214	0.013	0.134	0.912	0.049	0.054
E-2 Complications related to NBAL central venous catheterization	4.7	4.15	0.169	0.195	0.025	0.117	0.824	0.045	0.103

In the first round of the expert consensus, a total of nine experts proposed modifications. Six experts suggested changing “nursing document records” to “nursing documentation” to align with the expression specifications. Two experts pointed out that medication and NBAL were not included in the technical indicators for central venous catheter care. They recommended adding “safe medication” and “care of NBAL central venous catheter” to address this gap. One expert pointed out that the “Maintenance instructions for NBAL central venous catheters during the indwelling period” lacked nursing guidance after extubation. The following changes were agreed upon after discussion by the focus group: changing “A-11 nursing document” to “A-11 nursing documentation records,” adding “A-13 safe medication” and “B-12 NBAL central venous catheter care,” and revising “D-7 Maintenance instructions for NBAL central venous catheters during the indwelling period” to “Nursing guidance during NBAL central venous catheter placement and after extubation.” The values and coefficients of variation of the secondary indicator Ci in the second round are shown in Table 2.

**Table 2:** Ci values and variation coefficients of secondary indicators in the second round.

Technology	Evaluation indicator		After standardization		D+ (distance from the optimal solution)	D− (distance from the worst solution)	Ci (degree of proximity to the optimal solution)	CV	
	Importance	Complexity	Importance	Complexity				Importance	Complexity
	Mean	Mean	Mean	Mean					
A-1 Check	4.7	2.65	0.17	0.123	0.097	0.056	0.366	0.045	0.124
A-2 Postural management	3.95	2.6	0.143	0.12	0.105	0.029	0.216	0.088	0.169
A-3 Medical order processing	4.5	2.9	0.163	0.134	0.087	0.053	0.379	0.1	0.203
A-4 Condition assessment	4.8	4.45	0.174	0.206	0.014	0.113	0.89	0.033	0.101
A-5 Patient handover and transfer	4	3.05	0.145	0.141	0.085	0.043	0.336	0.1	0.212
A-6 basic daily care	3.2	2.45	0.116	0.113	0.123	0.004	0.031	0.081	0.101
A-7 Patient safety protection	4.5	3.25	0.163	0.15	0.071	0.062	0.466	0.078	0.119
A-8 Blood transfusion technology	4.7	3.55	0.17	0.164	0.056	0.077	0.579	0.066	0.098
A-9 Specimen collection	3.8	2.65	0.138	0.123	0.104	0.026	0.2	0.147	0.086
A-10 Prevention and control of hospital infection	4.45	3.5	0.161	0.162	0.06	0.066	0.524	0.101	0.129
A-11 Nursing documentation records	4.1	3.15	0.149	0.146	0.079	0.05	0.388	0.12	0.167
A-12 Emotional support	3.75	2.95	0.136	0.136	0.093	0.034	0.268	0.077	0.118
A-13 Safe medication	4.6	2.95	0.167	0.136	0.084	0.058	0.408	0.074	0.118
B-1 Environment and equipment preparation	4	2.6	0.145	0.12	0.105	0.031	0.228	0.125	0.131
B-2 NBAL equipment connection technology	4.6	3.85	0.167	0.178	0.043	0.086	0.667	0.096	0.319
B-3 NBAL pipeline pre-flushing technology	4.75	3.9	0.172	0.18	0.04	0.09	0.692	0.039	0.177
B-4 Measure leg circumference	3.5	2.35	0.127	0.109	0.121	0.011	0.083	0.071	0.097
B-5 Vascular access assessment and establishment	4.8	4.35	0.174	0.201	0.019	0.109	0.852	0.054	0.075
B-6 Connection to extracorporeal circulation	4.65	3.8	0.169	0.176	0.045	0.085	0.654	0.049	0.174
B-7 Setting treatment parameters	4.6	3.7	0.167	0.171	0.05	0.08	0.615	0.074	0.138
B-8 Heparinization of extracorporeal circulation circuit	4.8	3.8	0.174	0.176	0.044	0.087	0.664	0.054	0.121
B-9 ECG monitoring and oxygen inhalation	4.15	2.65	0.151	0.123	0.1	0.038	0.275	0.127	0.161
B-10 Blood flowing back into the body from the pipeline and turn off the machine	4.25	3.65	0.154	0.169	0.056	0.071	0.559	0.138	0.117
B-11 Medical waste disposal	3.7	2.45	0.134	0.113	0.115	0.018	0.135	0.273	0.101
B-12 NBAL central venous catheter care	4.55	3.8	0.165	0.176	0.045	0.083	0.648	0.076	0.2
C-1 Vital signs monitoring	4.65	2.75	0.169	0.127	0.093	0.056	0.376	0.049	0.105
C-2 Puncture point status	4.35	2.95	0.158	0.136	0.086	0.05	0.368	0.098	0.152
C-3 Pipeline condition	4.55	3.55	0.165	0.164	0.057	0.074	0.565	0.076	0.211
C-4 NBAL instrument parameters	4.65	3.7	0.169	0.171	0.049	0.082	0.626	0.07	0.3
C-5 Filter and adsorber condition monitoring	4.7	4.35	0.17	0.201	0.02	0.107	0.843	0.13	0.167
C-6 Dealing with NBAL instrument alarms	4.85	4.75	0.176	0.22	0	0.126	1	0.026	0.039
D-1 Explaining the coordination points in NBAL treatment	4.4	3.55	0.16	0.164	0.058	0.07	0.547	0.1	0.098
D-2 Inspection guidance	4	2.95	0.145	0.136	0.09	0.04	0.308	0.075	0.152
D-3 Safety guidance	4.4	3.05	0.16	0.141	0.081	0.054	0.4	0.1	0.18
D-4 Dietary guidance	3.8	2.75	0.138	0.127	0.1	0.028	0.219	0.095	0.141
D-5 Bed urination and defecation training	3.6	2.85	0.131	0.132	0.099	0.027	0.214	0.122	0.115
D-6 Ankle pump exercise	4	2.8	0.145	0.13	0.095	0.036	0.275	0.125	0.093
D-7 Nursing guidance during NBAL central venous catheter placement and after extubation	4.75	4.05	0.172	0.187	0.033	0.096	0.744	0.039	0.036
E-1 Complications of NBAL treatment	4.8	4.65	0.174	0.215	0.007	0.121	0.96	0.033	0.07
E-2 Complications related to NBAL central venous catheterization	4.65	4.4	0.168	0.204	0.018	0.109	0.865	0.049	0.055

**Figure 3:**
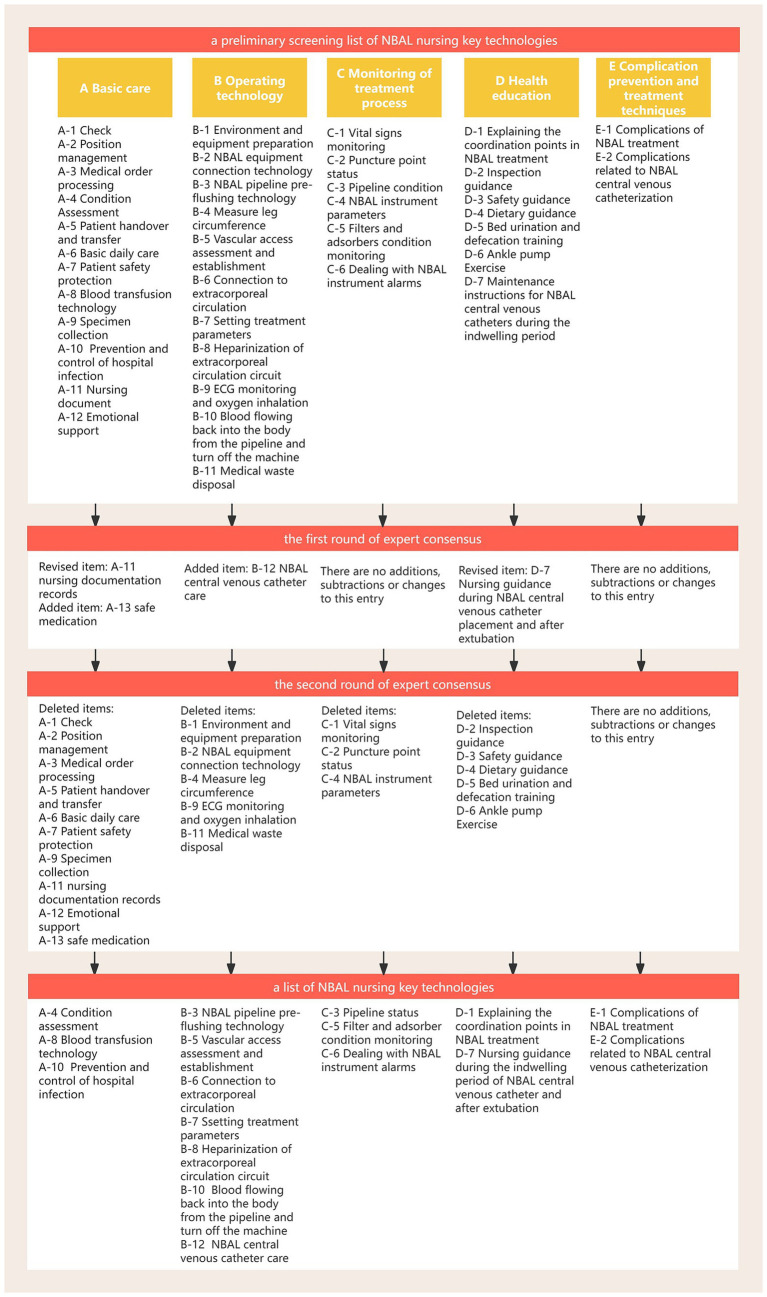
A flowchart showing the screening and evaluation process for key NBAL nursing technologies, along with the final selection of key techniques.

The following 22 secondary indicators had a coefficient of variation >0.25 and a mean value of importance or complexity of <3.5 in two rounds of expert consensus: A-1 check, A-2 position management, A-3 processing of medical orders, A-5 patient handover and transfer, A-6 basic daily care, A-7 patient safety protection, A-9 specimen collection, A-11 nursing documentation records, A-12 emotional support, B-1 environment and equipment preparation, B-2 NBAL equipment connection technology, B-4 measuring leg circumference, B-9 ECG monitoring and oxygen inhalation, B-11 medical waste disposal, C-1 vital sign monitoring, C-2 puncture point status, C-4 NBAL instrument parameters, D-2 inspection guidance, D-3 safety guidance, D-4 dietary guidance, D-5 bed urination and defecation training, and D-6 ankle pump exercise. This indicates that the degree of coordination among experts was insufficient and suggests that the importance or complexity of this nursing technology is relatively low. In addition, the complexity of the newly added A-13 safety medication was <3.5 in the second round of expert consensus. The ranking of these 23 technologies was also relatively low. The members of the research team discussed among themselves and reached a consensus that these 23 indicators cannot be used as key technologies for NBAL nursing, and thus, they were deleted. Figure 3 shows a flowchart of the screening and evaluation process for key NBAL nursing technologies, along with the final selection of key techniques. The 17 key NBAL nursing technologies finally selected are shown in Supplementary Table 3.

#### 3.3.2 Analytic hierarchy process results

AHP was employed to synthesize the evaluation outcomes from two rounds of expert correspondence regarding the first-level indicators. The relative importance of these indicators, listed in descending order, is as follows: B operating technology, C monitoring of treatment process, E complication prevention and treatment techniques, D health education, and A basic care in Table 3.

**Table 3:** Analytic hierarchy process results of first-level NBAL nursing indicators across two expert consultation rounds.

The first-level indicators	The first round	The second round
A basic care	0.043	0.038
B operating technology	0.411	0.414
C monitoring of treatment process	0.267	0.284
D health education	0.110	0.074
E complication prevention and treatment techniques	0.169	0.189

#### 3.3.3 Technique for order preference by similarity to ideal solution results

**Figure 4:**
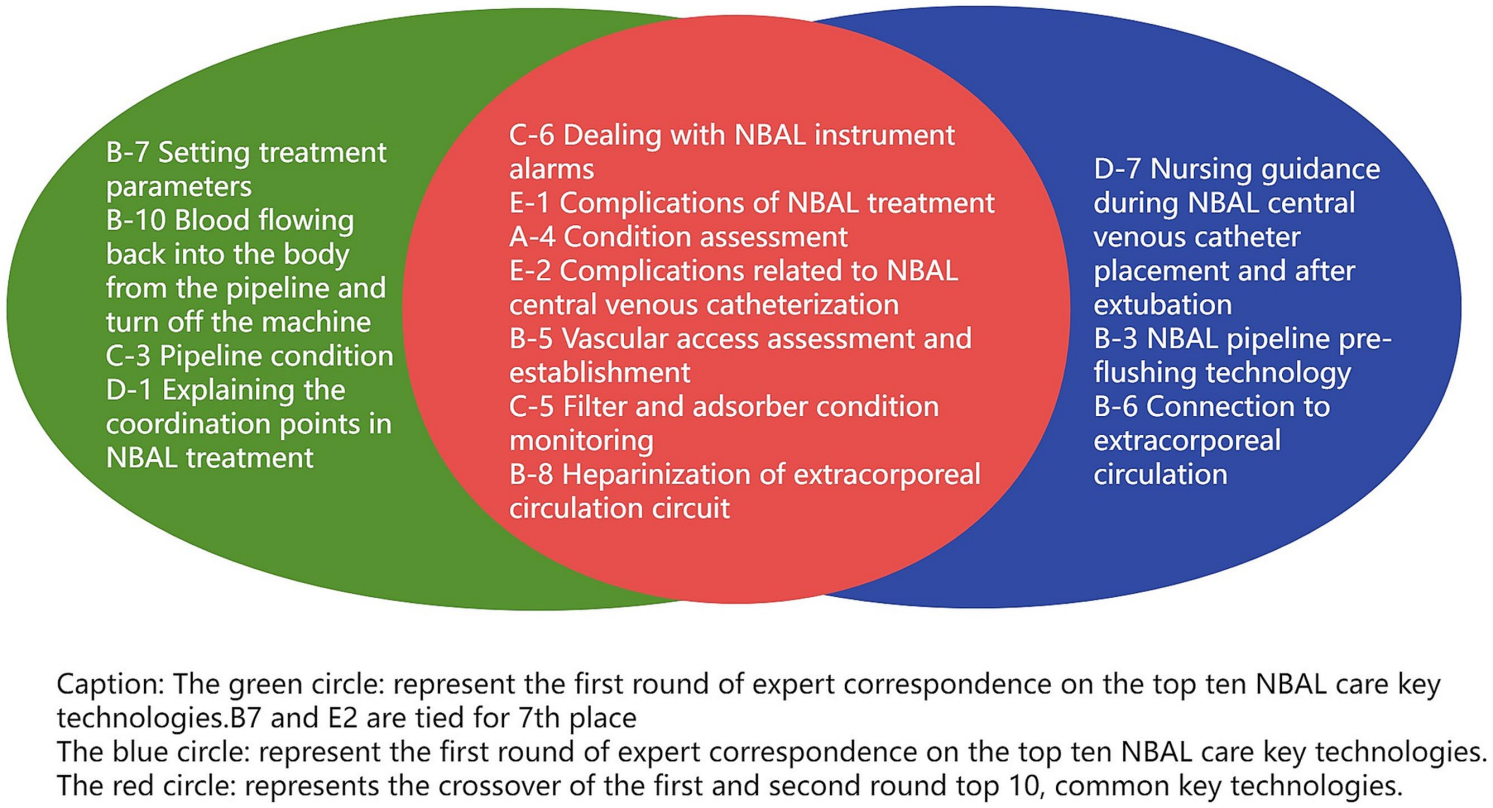
Application of the TOPSIS method to rank secondary indicators and identify the top 10 NBAL nursing key techniques from two rounds of expert consultation.

TOPSIS was utilized to rank the secondary indicators from two rounds of expert consultation. The resulting top 10 NBAL nursing key technologies are presented in Figure 4.

## 4 Discussion

### 4.1 The scientific nature of NBAL nursing key technology screening research methods

The Delphi method is the primary method for screening key technologies in NBAL nursing. The results can be considered relatively reliable only when the number of expert consensus inquiries is 15–50, the questionnaire response rate is ≥70%, and the expert authority coefficient is >0.70 (27). This study invited 20 experts from 17 provincial-level administrative regions across the country. The effective response rates of the questionnaires in the two rounds of consensus were 95 and 100%, respectively, and the expert authority levels were 0.910 and 0.930, respectively, thus meeting the implementation requirements of the Delphi method. After two rounds of expert consensus, the Kendall harmony coefficient of this study ranged from 0.402 to 0.620, upholding the consistency among expert opinions and desirability of the prediction results (28).

However, if only the Delphi expert consensus method is used, the promotion and application of the selected key technologies may be hindered by suitability issues. Examination of the screening research on key technologies globally makes it clear that the application of screening methods has evolved from the single Delphi expert consensus method to a composite method. Therefore, to create a preliminary screening list of key NBAL nursing technologies, this study initially used two methods, namely on-site investigation and literature research methods. Subsequently, the focus group discussion method was used to establish screening principles and evaluation indicators for key NBAL nursing technologies. The Delphi method was then used to consult experts through letters, which was followed by the application of the analytic hierarchy process and the TOPSIS comprehensive evaluation method to assess the key technologies of NBAL nursing. This approach ensured the scientific rigor of the screening results from both theoretical and practical perspectives.

### 4.2 Analysis of the importance of first-level indicators of NBAL nursing key technologies

For the primary indicators, it is evident from the results of the two rounds of expert consensus that *B: operation technology* and *C: treatment process monitoring technology* had the highest weights. The reasons are analyzed as follows: First, the NBAL treatment involves extracorporeal circulation (29), which means that blood is extracted from the body, processed by the NBAL device, and then reintroduced into the body. This process requires precise operation to ensure smooth and safe blood flow. Any operational errors can result in severe complications like blood coagulation, hemolysis, and infection, ultimately endangering the patient’s life. Second, the NBAL treatment inflicts certain damage on the patient’s body. For instance, NBAL necessitates deep vein intubation, which entails a prolonged indwelling period, and patients with liver failure often experience various complications, such as infection, bleeding, and deep vein thrombosis, because of coagulation disorders stemming from underlying diseases (30). Therefore, adherence to aseptic principles during the operation is crucial to minimize the risk of infection. Simultaneously, nurses must closely monitor the patient’s vital signs, including heart rate, blood pressure, pulse, and potential complications like bleeding, infection, and allergic reactions to promptly identify and prevent any adverse events. Third, the outcome and prognosis of the NBAL treatment are intricately linked to the patient’s physical condition and the recovery of liver function. Consequently, regular monitoring of the patient’s liver function indicators, electrolytes, and coagulation function, among other parameters, is essential during the NBAL treatment to assess the treatment’s efficacy and prognosis. In addition, nurses should pay close attention to the patient’s mental state and lifestyle habits, offer necessary psychological support and health guidance, and assist patients in better cooperating with the treatment and regaining their health. Taken together, operational technology and treatment process monitoring technology are pivotal in ensuring the safety and efficacy of the treatment. This aligns with the research findings of scholars like Dong (31) and Li (32). Thus, nurses must strictly adhere to operational protocols, closely monitor the patient’s vital signs and condition changes, and promptly address any abnormal situations to facilitate smooth progression of treatment and patient recovery.

*E: the weight of prevention and treatment technology for complications* is also important. The artificial liver support system is a treatment method based on blood purification and extracorporeal circulation. In most cases, the condition of the patients treated with artificial liver support has already descended to liver failure. Patients with liver failure often experience complications like coagulation dysfunction (33) and low immunity (34), making them more susceptible to complications during treatment. Severe cases can even be life-threatening. According to literature reports, the incidence rate of complications related to artificial liver support is 20.4–36.3% (35, 36). Therefore, it is crucial to have techniques in place to prevent and treat NBAL complications in patients undergoing artificial liver support treatment. Interestingly, *D: health education technology* and *A: basic nursing technology* are the least used in this treatment method. This may be because experts believe that these two technologies are relatively easy to learn and master and do not seem complicated in theory. However, both health education technology and basic nursing technology are essential throughout the NBAL treatment. Nurses still need to have excellent professional knowledge and skills to ensure the continuity, safety, and efficacy of the treatment.

Taken together, *B: operating techniques*, *C: treatment process monitoring techniques*, and *E: complication prevention techniques* are directly related to the life safety of NBAL patients and play a vital role in improving treatment effects, reducing complications, and enhancing the quality of life. Meanwhile, both *D: health education technology* and *A: basic nursing technology* are indispensable as NBAL nursing technology is an organic combination of five first-level indicators.

### 4.3 Analysis of the importance and complexity of secondary indicators of NBAL nursing key technologies

The NBAL treatment involves intricate technical connections and medical knowledge and is characterized by high professionalism, technical demands, risks, complications, challenging management, and complex patient conditions (37, 38). Therefore, special attention must be given to the 17 selected secondary indicators of NBAL nursing key technologies, with particular attention to the following indicators: C-6 dealing with NBAL instrument alarms, E-1 prevention and treatment of complications of NBAL treatment, A-4 condition assessment, E-2 prevention and treatment of complications related to NBAL central venous catheterization, B-5 vascular access assessment and establishment; C-5 filter and adsorber condition monitoring, and B-8 heparinization of extracorporeal circulation circuit. These seven technologies have all ranked among the top 10 in both rounds of sorting by expert consensus, which upholds their significance. The reasons and clinical implications are analyzed below:

First, the handling of C-6 dealing with NBAL instrument alarms is crucial.

An instrument alarm indicates that the data measured by the equipment may be inaccurate or unreliable, often because of instrument failure, abnormal operation, or changes in the patient’s vital signs. Failure to respond promptly or handle the alarm correctly can lead to several problems. These problems may include further damage to the equipment, potential safety hazards for patients’ treatment interruption or delay (which particularly impacts critically ill patients’ treatment outcomes), reduced work efficiency, increased workload for medical staff, and increased likelihood for doctors to reach an incorrect diagnosis or make incorrect treatment decisions, ultimately affecting patient outcomes.

Therefore, timely response and proper handling of instrument alarms are essential to ensure equipment stability, treatment efficacy, patient safety, medical staff efficiency, and overall high nursing service quality. However, our previous research has identified common difficulties and confusion among nurses when analyzing and responding to NBAL instrument alarms (16).

In clinical practice, improving the ability to respond to NBAL instrument alarms can be achieved through various strategies:

- Establish an instrument alarm management team comprising doctors, head nurses, artificial liver specialist nurses, and manufacturer engineers, among other relevant personnel. Develop different response and treatment processes for the different causes of instrument alarms. Regularly evaluate and update response plans for adaptability and efficacy.
- Invite instrument and equipment experts to conduct regular training for nurses to ensure they are familiar with the equipment’s structure, working principle, common alarm causes, and solutions. Organize recurrent training to keep nursing staff’s knowledge and skills aligned with equipment requirements.
- Implement a regular maintenance schedule for instruments. Proper maintenance can extend the equipment’s service life and reduce failure rates (39). Maintain records of maintenance activities in the artificial liver chamber.
- Conduct regular simulation drills for NBAL instrument alarms. Collaborate with other medical institutions using similar equipment to share experiences and lessons learned.

Second, E-1 Prevention and treatment of complications of the NBAL treatment.

E-1: *The prevention and treatment of complications of the NBAL treatment* is particularly important. Nurses must be knowledgeable about the complications associated with NBAL treatment, including the causes and frequency of complications related to NBAL central venous catheterization. This knowledge will enable them to identify, prevent, and treat these complications promptly.

Third, A-4 condition assessment.

Meticulous observation of the patient’s condition is essential for timely detection and prediction of changes, thus enabling precise care. Medical staff must thoroughly assess the patient during the peri-treatment period of NBAL, closely monitoring the patient’s vital signs, oxygen saturation, blood sugar, coagulation function, and other parameters. They should also monitor the patient’s consciousness, pupils (the shape, symmetry, size, and response to light of the pupils on both sides), complexion, skin temperature, and limb activities. In addition, accurate recording of the patient’s urine output, subcutaneous symptoms, nausea and vomiting, and any abnormalities is crucial. Any anomalies noted should be promptly reported to the doctor.

Fourth, E-2 Prevention and treatment of complications related to NBAL central venous catheterization.

Complications associated with NBAL central venous catheterization can lead to extended hospital stays, higher medical costs, added financial stress, and increased psychological pressure on both patients and their families. These complications can also significantly impact the efficacy of the treatment.

Fifth, B-5 vascular access assessment and establishment.

It is a prerequisite for patients to undergo the NBAL treatment. To meet the blood flow requirements for undergoing NBAL treatment, the femoral vein, subclavian vein, or internal jugular vein can be selected as the catheter insertion site. The advantages of selecting the subclavian vein are that its position is relatively fixed, the puncture site is easy to keep clean, and it is less likely to cause catheter-related bloodstream infection (CRBSI) (40). Furthermore, it has a long retention time and does not affect the patient’s activities. However, its disadvantage is that it is easily compressed by the clavicle. Owing to lumen stenosis, the risk of thrombosis is higher in the subclavian vein than in the other veins, and the technique of subclavian vein catheterization is difficult. The effect of compression hemostasis is poor, leading to many bleeding complications (41). Although the internal jugular venous catheter does not have these disadvantages and imposes fewer restrictions on the patient’s activities, its disadvantage is the relatively high incidence of CRBSI. The advantages of femoral vein catheterization include a good compression and hemostasis effect, low incidence of hematoma, no higher incidence of CRBSI when compared with the internal jugular vein, convenient puncture, and low technical requirements (42). Indwelling double-lumen or triple-lumen catheters in the femoral vein is safer and more reliable for patients with liver failure and poor coagulation function. Therefore, we recommend femoral vein as the first choice for catheter placement in patients with liver failure undergoing NBAL treatment, followed by the internal jugular vein. We recommend avoiding catheter placement in subclavian vein placement.

Sixth, C-5 filter and adsorber condition monitoring.

The choice of filters and adsorbers directly impacts the efficacy of removing harmful substances from the patient’s body. Medical staff are encouraged to strictly follow medical advice and instructions when using consumables. During the NBAL treatment, it is important to closely monitor the filters and adsorbers for any abnormalities, such as coagulation or hemolysis, and to promptly replace them if any issues are identified.

Seventh, B-8 heparinization of extracorporeal circulation circuit.

Heparinization is a critical step in the NBAL extracorporeal circulation process. It can prevent blood coagulation, maintain blood fluidity, prevent thrombi or clots from forming in the pipeline, ensure the efficacy of extracorporeal circulation, and prolong the service life of extracorporeal circulation pipelines. This reduces the frequency of pipeline blockage or replacement because of coagulation and minimizes the waste of medical resources. Therefore, when selecting anticoagulants, factors like anticoagulant effect, safety, stability, and cost-effectiveness should be considered. Regarding the timing of coagulation monitoring, patients are recommended to monitor their coagulation status dynamically before, during, and after NBAL treatment. This approach allows for individualized use of anticoagulants.

As shown in Figure 3, that the following technologies failed to enter the top 10 in the secondary indicator ranking of NBAL nursing key technologies (A-8 blood transfusion technology, A-10 prevention and control of hospital infection, B-3 NBAL pipeline pre-flushing technology, B-6 connection to extracorporeal circulation, B-7 setting treatment parameters, B-10 blood flowing back into the body from the pipeline and turn off the machine, B-12 NBAL central venous catheter care, C-3 pipeline status, D-1 explaining the coordination points in NBAL treatment, and D-7 maintenance instructions for NBAL central venous catheters during the indwelling period). These technologies are important in the NBAL treatment; however, they are relatively easy to learn and master in the clinical practice of the NBAL treatment and their operation is less difficult and requires lower proficiency and quality of nursing staff; thus, their CI value is lower.

## 5 Conclusion

In this study, we defined the concept and significance of NBAL nursing key technologies, and through a series of empirical research methods, we ultimately identified 17 NBAL nursing key technologies across five categories. These technologies included seven items of operational technology, three items of basic nursing technology, three items of treatment process monitoring technology, two items of health education technology, and two items of complication prevention and treatment technology. The resultant key technologies, which encompass diverse nursing processes, are distinguished by their comprehensiveness, specificity, and practical applicability. These technologies not only elevate the quality of nursing care and enhance therapeutic outcomes associated with NBAL interventions but also offer a crucial theoretical foundation and practical guidance for the advancement of the standardization and professionalization of abiotic artificial liver care.

Notably, this study has some limitations. First, due to constrains in resources of manpower and time, only 17 provincial-level administrative regions were included in the consensus consultation, which may not be representative of the entire country. To address this, it is essential to widen the range of consultation experts to ensure a more comprehensive perspective. Second, although we identified key technologies for NBAL nursing, further refinement and standardization of secondary indicators are needed to establish a standardized operational process for NBAL nursing technology. In the future, the focus group plans to implement the findings in clinical settings to assess their impact on reducing complications, enhancing treatment outcomes and quality of care, and ultimately improving patients’ quality of life.

## Data Availability

The original contributions presented in the study are included in the article/Supplementary material, further inquiries can be directed to the corresponding author.
