# Supplementary material for: Screening and evaluation of key technologies for non-bioartificial liver care: an empirical study
Source: Front Med (Lausanne). 2025 Jan 7;11:1459428. doi: 10.3389/fmed.2024.1459428 (PMC11745882; doi:10.3389/fmed.2024.1459428)
Supplement: Supplementary file 1 [file Table_1.DOCX]

**Supplementary Table 1**. Initial screening technology list of key NBAL nursing technologies

| First level indicator | Second level indicator |
| --- | --- |
| A Basic care | A-1 check, A-2 position management, A-3 medical order processing, A-4 condition assessment, A-5 patient handover and transfer, A-6 basic daily care, A-7 patient safety protection, A-8 blood transfusion technology, A-9 specimen collection, A-10 prevention and control of hospital infection, A -11 nursing document, and A -12 emotional support |
| B Operation technology | B-1 environment and equipment preparation, B-2 NBAL equipment connection technology, B-3 NBAL pipeline pre-flushing technology, B-4 measuring leg circumference, B-5 vascular access assessment and establishment, B-6 connection to extracorporeal circulation, B-7 setting treatment parameters, B-8 heparinization of extracorporeal circulation circuit, B-9 ECG monitoring and oxygen inhalation, B-10 blood flowing back into the body from the pipeline and turn off the machine, and B-11 medical waste disposal |
| C Treatment process monitoring | C-1 vital sign monitoring, C-2 puncture point status, C-3 pipeline status, C-4 NBAL instrument parameters, C-5 filter and adsorber condition monitoring, and C-6 Dealing with NBAL instrument alarms |
| D Health education | D-1 explaining the coordination points in NBAL treatment, D-2 inspection guidance, D-3 safety guidance, D-4 dietary guidance, D-5 bed urination and defecation training, D-6 ankle pump exercise, and D-7 maintenance instructions for NBAL central venous catheters during the indwelling period |
| E Prevention of complications | E-1 complications of NBAL treatment and E-2 complications related to NBAL central venous catheterization |

**Supplementary Table 2**. Basic information on consensus with experts

| Category | Group | Number of people | Composition ratio (%) |
| --- | --- | --- | --- |
| Gender | Male | 5 | 25 |
|  | Female | 15 | 75 |
| Age (years) | 30-39 | 3 | 15 |
|  | 40-49 | 13 | 65 |
|  | ≥50 | 4 | 20 |
| Educational qualifications | Undergraduate | 10 | 50 |
|  | Master | 8 | 40 |
|  | PhD | 2 | 10 |
| Professional Title | Intermediate | 2 | 10 |
|  | Associate senior | 13 | 65 |
|  | Senior | 5 | 25 |
| Position | Director of Nursing Department | 1 | 5 |
|  | Head nurse | 12 | 60 |
|  | Clinical nurse | 5 | 25 |
|  | Clinicians | 2 | 10 |
| Working hours (years) | 10-19 | 3 | 15 |
|  | 20-29 | 12 | 60 |
|  | ≥30 | 5 | 25 |
| *: A total of two rounds of expert consensus were conducted. In each round, 20 questionnaires were distributed, 20 were recovered, and 20 were valid questionnaires. | | | |

**Supplementary Table 3**. Key NBAL nursing technologies selected after two rounds of expert consensus

| First level indicator | Secondary indicators |
| --- | --- |
| A Basic care | A-4 Condition assessment |
|  | A-8 Blood transfusion technology |
|  | A-10 Prevention and control of hospital infection |
| B Operating technology | B-3 NBAL pipeline pre-flushing technology |
|  | B-5 Vascular access assessment and establishment |
|  | B-6 Connection to extracorporeal circulation |
|  | B-7 Setting treatment parameters |
|  | B-8 Heparinization of extracorporeal circulation circuit |
|  | B-10 Blood flowing back into the body from the pipeline and turn off the machine |
|  | B-12 NBAL central venous catheter care |
| C Monitoring of treatment process | C-3 Pipeline status |
|  | C-5 Filter and adsorber condition monitoring |
|  | C-6 Dealing with NBAL instrument alarms |
| D Health education | D-1 Explaining the coordination points in NBAL treatment |
|  | D-7 Nursing guidance during the indwelling period of NBAL central venous catheter and after extubation |
| E Complication prevention and treatment techniques | E-1 Complications of NBAL treatment |
|  | E-2 Complications related to NBAL central venous catheterization |
